# Decoding ferroptosis via integrated multi‐omics analyses in rice blast

**DOI:** 10.1002/imt2.70178

**Published:** 2026-10-07

**Authors:** Zhibin Liang, Qing Shen, Nam Khoa Nguyen, Ruhui Long, Yuming Ma, Qiao Liu, Naweed I. Naqvi, Nam‐Soo Jwa, Yi‐Zhen Deng

**Affiliations:** ^1^ Guangdong Basic Research Center of Excellence for Precise Breeding of Future Crops, Integrative Microbiology Research Centre South China Agricultural University Guangzhou China; ^2^ Temasek Life Sciences Laboratory Singapore Singapore; ^3^ Division of Integrative Bioscience and Biotechnology, College of Life Sciences Sejong University Seoul Republic of Korea; ^4^ Department of Biological Sciences National University of Singapore Singapore Singapore

## Abstract

The rice‐*Magnaporthe oryzae* pathosystem is presented as a host‐pathogen ferroptosis model. Multi‐omics insights connect ferroptosis to several cellular processes, such as iron homeostasis, reactive oxygen species (ROS)/redox imbalance, and polyunsaturated fatty acid‐containing phospholipid (PUFA‐PL) peroxidation, under multiple regulators in rice and *M. oryzae*, respectively. Interestingly, ferroptosis drives two opposite biological outcomes: fungal infection‐related development and rice immunity.

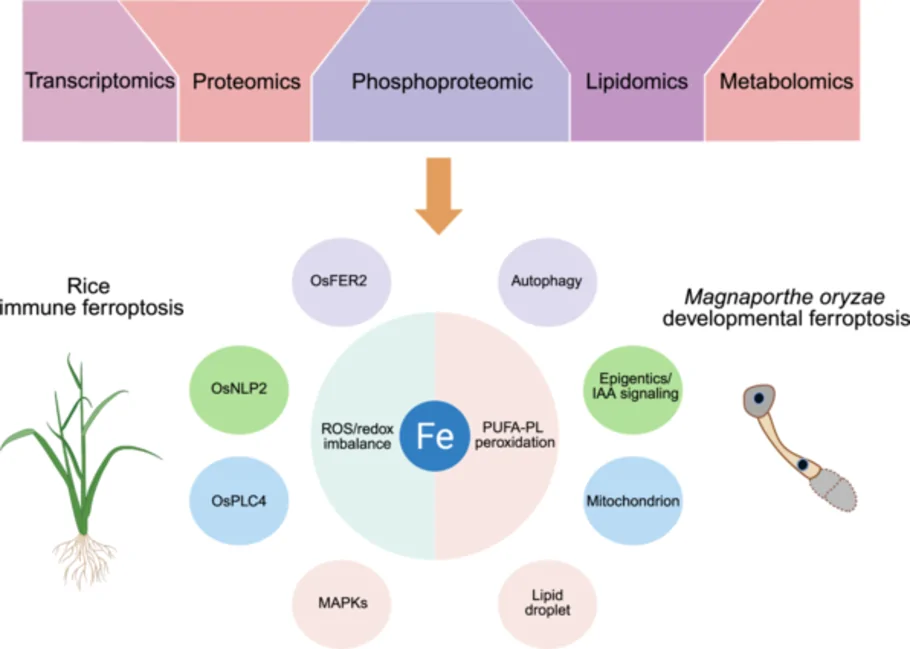

Ferroptosis is classically defined as an iron‐dependent form of regulated cell death driven by the accumulation of lethal lipid peroxides. In mammalian cells, the glutathione peroxidase 4‐glutathione (GPX4‐GSH) and ferroptosis suppressor protein 1‐coenzyme Q (FSP1‐CoQ) axes are well‐characterized protective systems that restrain lipid peroxidation [1]. However, counterpart canonical GPX4‐ or FSP1‐centered systems are absent in plants or fungi. Instead, plant or fungal ferroptosis depends on convergent biochemical, pharmacological, genetic, and omics evidence.

The rice–*Magnaporthe oryzae* interaction is a robust model system for examining ferroptosis across the host‐pathogen interface. In rice, avirulent *M. oryzae* infection induces immune cell death characteristics of ferroptosis, including iron accumulation, reactive oxygen species (ROS) production, and lipid peroxidation, and sensitivity to ferroptosis‐inhibitory treatments [2]. In *M. oryzae*, ferroptosis contributes to conidial cell death and infection‐related development, thereby supporting fungal pathogenicity [3]. Thus, the same chemical logic—iron accumulation, ROS/redox imbalance, and lipid peroxidation can produce opposite biological outcomes depending on the organism, developmental stage, and infection context.

In this Commentary, we use multi‐omics insights from rice–*M. oryzae* interaction to build a module‐level framework that connects specific data layers with ferroptosis‐associated processes and outcomes. Transcriptomics and proteomics helped identify infection stage‐specific candidate genetic regulators. Phosphoproteomics and acetylomics capture signaling and post‐translational regulators. Lipidomics defines lipid‐substrate remodeling. Metabolomics/metallomics together with imaging‐based readouts link iron, ROS, lipid peroxidation, and cell‐death phenotypes at infection sites. By integrating these layers with genetic and pharmacological perturbations, this framework separates correlation‐level omics signatures from causality‐supported ferroptosis mechanisms and highlights strategies to suppress pathogen‐beneficial ferroptosis while tuning host‐beneficial immune ferroptosis. Overall, these multi‐omics analyses reveal the integrative signaling pathway consisting of OsMEK2‐OsMPK1, OsFER2, OsNLP2, cytosolic Ca^2+^ influx, and OsPLC4 that regulate rice immune ferroptosis [4, 5, 6, 7, 8, 9]; as well as linking developmental ferroptosis to MoFpn1/autophagy, polyunsaturated fatty acid‐containing phospholipids (PUFA‐PLs), phosphatidylethanolamines, NADPH oxidase (Nox)/lipid oxidation, Gcn5‐dependent acetylation, histone deacetylase (HDAC)‐associated transcriptional programs, and fungal indole‐3‐acetic acid (IAA) metabolism, on the pathogen side [10, 11, 12, 13, 14, 15, 16, 17, 18].

## OPERATIONAL DEFINITION AND OMICS LOGIC

Since the well‐established canonical GPX4/FSP1‐centered markers in mammalian cells are not available for plants and fungi, evidence strength becomes central to functional classification. We use “ferroptosis” for cases in which iron dependency, lipid peroxidation, pharmacological suppression by lipid radical‐trapping antioxidants or iron chelators, and genetic or biochemical perturbation converge. And we use “ferroptosis‐like cell death” when iron/ROS/lipid‐peroxidation features are present, but genetic, lipidomic, or perturbation‐based evidence remains incomplete. On this basis, rice immune ferroptosis and *M. oryzae* developmental ferroptosis are viewed as ferroptosis, not merely as ROS‐ and iron‐associated cell death.

This classification matters because ROS and iron accumulation, as well as cell‐death phenotypes can accompany not only ferroptosis but also other stress or defense programs. Ferroptosis is tightly coupled with the hypersensitive response (HR) triggered by effector‐triggered immunity (ETI) during the incompatible interaction between rice and *M*. *oryzae* [2]. HR represents the visible cell‐death phenotype, while ferroptosis contributes to HR‐associated cell death in the rice–*M. oryzae* interaction. Both processes rely on sustained cytoplasmic Ca^2+^ influx, massive ROS bursts, and GSH depletion [7, 8, 9]. Autophagy does regulate ferroptosis, particularly during fungal pathogenic development, but autophagy constitutes a separate biological process distinct from ferroptosis. Pharmacological agents such as ferrostatin‐1 (Fer‐1), liproxstatin‐1 (Lip‐1), deferoxamine (DFO), ciclopirox olamine (CPX), diphenyleneiodonium (DPI), erastin, ethylene glycol‐bis(2‐aminoethylether)‐N,N,N′,N′‐tetra‐acetic acid (EGTA), trifluoperazine hydrochloride (TFP), and asibenzolar‐*S*‐methyl (ASM) remain valuable, especially when combined with genetic perturbation, biochemical readouts, and omics‐supported regulation. Such an evidence‐based definition for ferroptosis is used throughout the study and summarized in Table S1, with corresponding references provided in Table S8.

The omics layers are not equivalent. Transcriptomics identifies infection‐stage programs and candidate gene regulators, but by itself, it usually provides correlation‐level evidence. Proteomics adds information on protein abundance, turnover, and localization‐linked regulation. Phosphoproteomics and acetylomics better capture signaling and post‐translational controls. Lipidomics insights are closest to ferroptosis execution among all the omics methods discussed in this Commentary, because ferroptosis is driven by membrane lipid peroxidation. Metabolomics and metallomics/ionomics measure Fe^2+^/Fe^3+^, GSH, malondialdehyde (MDA), IAA, Ca^2+^, and related redox or metal pools, although these markers become specific or relevant only when connected to lipid oxidation and perturbation. Targeted biochemical or imaging‐based readouts, including MDA quantification, 3,3′‐diaminobenzidine (DAB) staining, Prussian blue staining, histone H1 labeling, C11‐BODIPY 581/591 staining, and Calcein‐AM staining, provide spatial or functional validation at infection sites. In short, transcriptomics and proteomics nominate modules, whereas lipidomics and metallomics insights define biochemical state, and genetic or pharmacological perturbation establishes causality for ferroptosis.

On this basis, we summarize a module‐level framework integrating rice and pathogen ferroptosis, as illustrated in Figure 1. In this framework, five modules are especially informative: (i) iron homeostasis/metallomics, (ii) ROS/redox and antioxidant balance, (iii) lipid‐substrate and lipid‐peroxidation chemistry, (iv) Ca^2+^/mitogen‐activated protein kinase (MAPK)/phospholipid signaling, and (v) autophagy/organelle‐associated remodeling. In the following sections, we discuss such omics‐dissected ferroptosis in *M. oryzae* pathogenicity and rice immunity, respectively.

**FIGURE 1:**
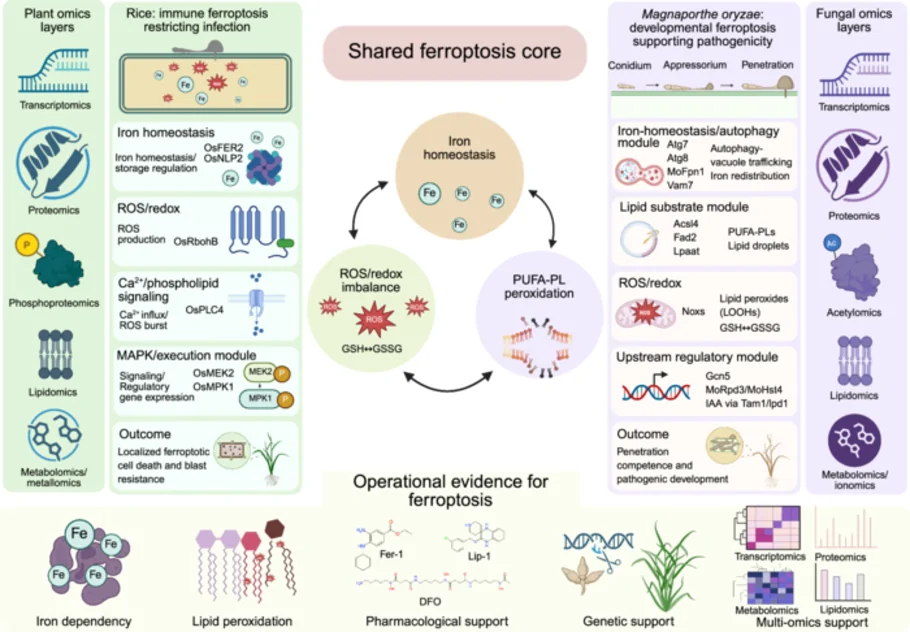
Omics‐dissected dual ferroptosis in the rice–*M. oryzae* pathosystem. The rice‐*M. oryzae* interaction provides a dual host‐pathogen model in which ferroptosis contributes to opposite biological outcomes. In rice, immune ferroptosis restricts infection through coordinated iron‐homeostasis regulation, ROS/redox amplification, Ca^2+^/phospholipid signaling, and MAPK‐associated immune execution. Representative rice regulators include OsFER2 and OsNLP2 for iron‐homeostasis regulation, OsRbohB for ROS production, OsPLC4 for Ca^2+^/phospholipid‐linked ROS burst, and OsMEK2‐OsMPK1 for MAPK‐associated ferroptotic cell death. In *M. oryzae*, developmental ferroptosis supports pathogenic development by promoting appressorium maturation, penetration competence, and infection‐related growth. Fungal modules include iron‐homeostasis/autophagy remodeling mediated by Atg7, Atg8, MoFpn1, and Vam7; lipid substrate remodeling involving the lipid metabolism enzymes Acsl4, Fad2, and Lpaat, the lipid substrates PUFA‐PLs, and the organelle lipid droplets; ROS/redox regulation through Noxs, lipid hydroperoxides, and GSH/GSSG balance; and upstream regulatory inputs including Gcn5‐dependent acetylation, MoRpd3/MoHst4‐linked transcriptional regulation, and IAA biosynthesis via Tam1/Ipd1. The shared ferroptosis core integrates iron homeostasis, ROS/redox imbalance, and PUFA‐PL peroxidation as the convergent biochemical basis of ferroptotic execution. Plant and fungal omics sidebars indicate the major evidence layers used to dissect these modules, including transcriptomics, proteomics, phosphoproteomics, acetylomics, lipidomics, metabolomics, metallomics, or ionomics. The bottom panel summarizes operational evidence used to define ferroptosis‐related cell death in plant and fungal systems, including iron dependency, lipid peroxidation, pharmacological support, genetic support, and multi‐omics‐supported regulation. Created with BioRender.com. IAA, indole‐3‐acetic acid; MAPK, mitogen‐activated protein kinase; PUFA‐PL, polyunsaturated fatty acid‐containing phospholipid; ROS, reactive oxygen species.

## OMICS‐DISSECTED FERROPTOSIS IN *M. ORYZAE* PATHOGENICITY

In *M. oryzae*, ferroptosis is viewed as a developmental module that contributes to conidial cell death and infection‐related morphogenesis rather than as a passive terminal event. Conidial ferroptosis is suppressed by iron chelation, antioxidants, and ferroptosis inhibitors, while being promoted by iron or oxidative stress [3]. This established a functional link between ferroptosis and fungal pathogenic development. Later omics‐oriented research showed that this process depends on coordinated regulation of iron transport, autophagy, organelles, lipid substrates, and metabolic inputs.

The study of *M. oryzae* Ferroportin 1 (MoFpn1) illustrated why transcript abundance alone is not sufficient as a readout for function. MoFpn1 is an important efflux pump for intracellular iron. Loss of MoFpn1, or its degradation via autophagy, results in higher accumulation of intracellular iron. MoFpn1 interacts with Atg8 and the Vam7 SNARE complex, and the autophagy‐dependent delivery of MoFpn1 from the membrane to the vacuole enables the iron increase required for timely ferroptosis and pathogenic development in *M. oryzae* [10]. This mechanism is primarily a protein‐localization and turnover problem, thus rendering proteomics, cell biology, and targeted perturbation as essential tools for understanding ferroptosis regulation.

The lipid peroxides are positioned at the execution layer of ferroptosis. Acsl4 (acyl‐CoA synthetase long‐chain family) and Fad2 (fatty acid desaturase) contribute to the biosynthesis of PUFA‐PLs that provide lipid‐peroxidation‐prone substrates [13]. Lpaat (lysophosphatidic acid acyltransferase) acts through phosphatidylethanolamine metabolism and links lipid droplets, autophagy, and ferroptosis‐associated appressorium development [14]. These studies provided a broader functional framework rather than a single generic lipid statement: Acsl4/Fad2 and Lpaat converge on lipid‐peroxidation competence, but they do so partly through distinct lipid classes and developmental contexts. Lipidomics, therefore, directly helped test whether fungal cells contain ferroptosis‐enabling lipid substrates.

Upstream regulation adds another omics dimension. Autophagy‐driven mitochondrial degradation was found essential for ferroptosis, unveiling the organelle‐associated regulation and metabolic functions in such cell death [11]. An iron–copper chaperone Ict1 controls ferroptosis by regulating conidial iron accumulation, redox homeostasis, and mitophagy [12]. Gcn5‐dependent acetylation affects Atg7 activity that is required to induce autophagy [15, 16], whereas HDAC–complexes containing MoRpd3/MoHst4 likely influence conidial cell death states too [17]. Since the ferroptosis role of MoRpd3 is less established than the Gcn5‐Atg7 acetylome/autophagy link, it is better framed as an upstream developmental and epigenetic regulator rather than as a proven ferroptosis executor. Fungal IAA biosynthesis through Tam1 and Ipd1 provides another case of metabolic regulation of iron accumulation, lipid oxidation, and autophagy during conidial cell death and pathogenic development in *M. oryzae* [18].

## OMICS‐DISSECTED FERROPTOSIS IN RICE IMMUNITY

In rice, ferroptosis was first defined through infection‐site chemistry and pharmacology. During incompatible rice–*M. oryzae* interaction, intracellular ferric iron accumulation, ROS production, and lipid peroxidation were identified as executors of ferroptotic cell death during the rice HR in host plants. OsRbohB, a respiratory burst oxidase homolog, generates the specific ROS moieties that enable such incompatible interaction during ETI [2]. Such HR cell death could be suppressed by ferroptosis‐inhibitory treatments [2], supporting that it is ferroptotic cell death. Functional and spatial evidence is needed to confirm the exact role of ferroptosis in rice immunity, while comprehensive lipidomics and metallomics analyses at infection sites remain lacking.

Subsequent rice studies mapped upstream regulators of such immunity‐associated ferroptosis module. OsMEK2‐OsMPK1 signaling positively regulates ferroptotic cell death and defense responses [4], and subsequent research connected OsMEK2 with cytosolic Ca^2+^ influx and ferroptotic immune outputs therein [8]. OsFER2, a ferritin‐related iron‐homeostasis protein, positively regulates ferroptotic cell death and resistance against *M. oryzae* [5]. In contrast, OsNLP2 acts as a negative regulator of ferroptosis and host immunity through the restriction of iron/ROS amplification [6].

Calcium and phospholipid signaling represent another key regulatory node in rice. Cytoplasmic Ca^2+^ influx mediates iron‐ and ROS‐dependent ferroptotic cell death during rice immunity [7]. OsPLC4 enhances Ca^2+^ and ROS bursts during ETI, thereby promoting ferroptotic cell death [9]. These studies support a Ca^2+^/MAPK/phospholipid‐signaling module that links upstream immune signaling to iron‐ROS‐lipid peroxidation, pointing to a network architecture instead of a simple linear signaling cascade.

## MODULE‐LEVEL MULTI‐OMICS INTEGRATION

The aforementioned regulatory modules in *M. oryzae* or rice ferroptosis were usually identified based on experimental evidence and validation, while omics resources in the public domain place such regulators into broader infection‐stage networks. Figure 2A functions as the representative data‐input summary panel. On the host side, rice proteome/phosphoproteome/acetylome dataset MSV000099743/PXD070288 (Tables S2 and S3) could be used to generate a ferroptosis‐module proteome map across 12, 24, and 48 h post‐inoculation (hpi). This map is paired with representative targeted infection‐site DAB staining, Prussian blue staining, and ferroptotic cell‐death images (Figure 2A). On the pathogen side, omics methods including transcriptomics (PRJEB45007), proteomics [15], and acetylomics [16] enabled systematic identification of *M. oryzae* ferroptosis regulators. Atg7, Atg8, and MoFpn1 are representative regulators displaying multi‐layered molecular changes during autophagy‐regulated ferroptosis. Lipidomics analyses unveiled altered lipid profiles controlled by specific metabolic enzymes Acsl4, Fad2, and Lpaat during ferroptosis‐dependent conidial pathogenic development. Representative targeted imaging‐based readouts include C11‐BODIPY 581/591 and Calcein‐AM staining, and histone H1‐RFP labeling. These microscopic analyses reveal lipid peroxidation and the resultant ferroptotic cell‐death dynamics during infection‐associated morphogenesis and development in *M. oryzae*. The detailed interpretation, source datasets, and supporting references are provided in Tables S2–S5.

**FIGURE 2:**
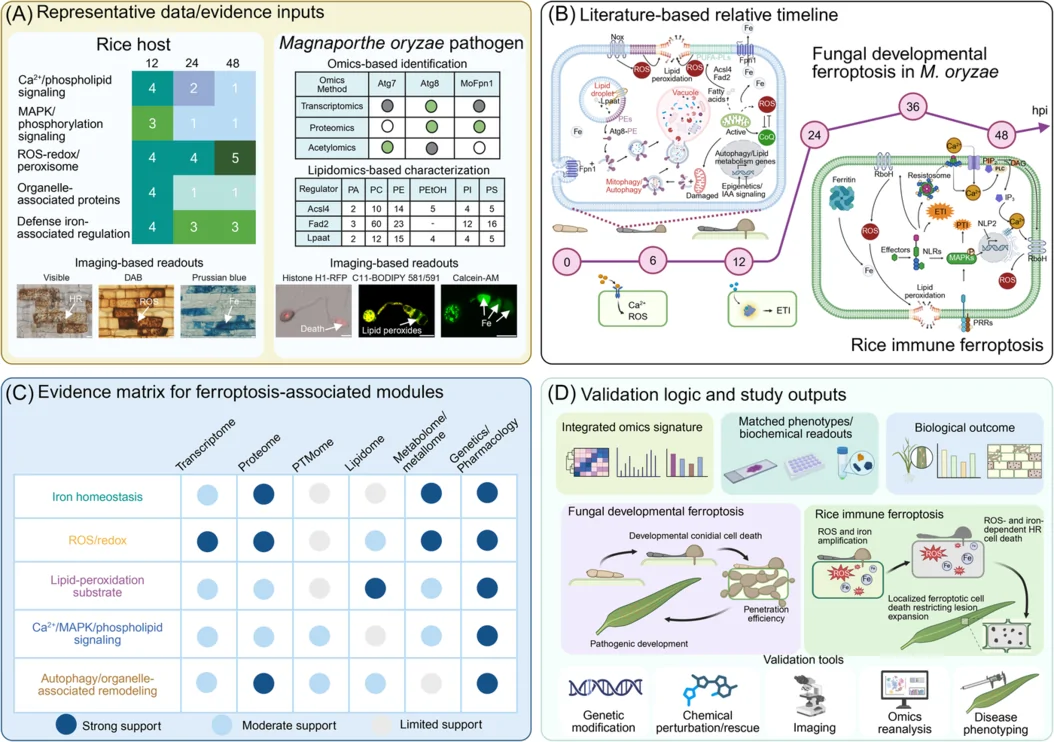
Data‐backed multi‐omics framework for resolving ferroptosis in the rice–*M. oryzae* pathosystem. (A) Representative data/evidence inputs for ferroptosis‐associated module analysis. Integrative rice proteome/phosphoproteome/acetylome resource MSV000099743/PXD070288 gives rise to the ferroptosis‐associated features across 12, 24, and 48 h post‐inoculation (hpi), on the rice host side. The score scale and supporting criteria are provided in Table S2. Module support score description: 1−5, from low to high. Representative rice targeted imaging‐based readouts include visible HR‐like symptoms, DAB staining for ROS accumulation, and Prussian blue staining for iron deposition during incompatible rice–*M. oryzae* interaction. In *M. oryzae* pathogen side, Atg7, Atg8, and MoFpn1 were established as ferroptosis regulators based on omics methods, including transcriptomics, proteomics, and acetylomics. Green circles represent omics methods capable of analyzing these regulatory factors, while gray and white circles denote omics approaches that are either incompatible with this type of analysis or require additional downstream analysis, respectively. Lipidomics profiling was used to screen for ferroptosis‐associated lipid species by comparing lipid abundance between wild‐type and mutants of lipid metabolic enzymes. “‐” indicates significant changes were not detected. Targeted imaging‐based assays for analyzing *M. oryzae* ferroptosis during conidial development include histone H1‐RFP labeling to assess *M. oryzae* conidial cell death, C11‐BODIPY 581/591 staining for lipid peroxides, and Calcein‐AM staining for visualization of iron (Fe). The scale bars for the imaging‐based readouts are 10 µm. Interpretation and source data are provided in Tables S3−S5. (B) Literature‐based relative host‐pathogen timeline shows the temporal relationship between fungal developmental ferroptosis and rice immune ferroptosis. On the fungal side, ferroptosis‐associated developmental processes are linked to conidial cell death, appressorium maturation, penetration competence, and early invasive growth. On the rice side, immune ferroptosis is represented as a progressive process involving early Ca^2+^/ROS signaling, iron accumulation, ROS amplification, lipid peroxidation, and localized ferroptotic cell death. The time points shown provide a relative framework based on published studies and should not be interpreted as a universal timeline for all rice‐*M. oryzae* experimental conditions. (C) Evidence matrix for ferroptosis‐associated modules across transcriptome, proteome, post‐translational modification (PTMome; phosphorylation and acetylation), lipidome, metabolome/metallome, and genetics/pharmacology evidence layers. Dark blue, light blue, and gray circles indicate strong, moderate, and limited support, respectively. Evidence strength was assigned using a semi‐quantitative rubric based on the directness of the readout, relevance to infection‐stage ferroptosis, and support from genetic or pharmacological perturbation. Detailed scoring criteria and source evidence are provided in Tables S6 and S7. (D) Validation logic and study outputs linking integrated omics signatures to matched biochemical, imaging, and phenotypic readouts. For fungal developmental ferroptosis, validation outputs include developmental conidial cell death, appressorium maturation, penetration efficiency, and pathogenic development. For rice immune ferroptosis, validation outputs include ROS and iron amplification, lipid peroxidation, localized ferroptotic cell death, and restricted lesion expansion. Genetic perturbation, chemical perturbation/rescue, imaging, omics analysis or reanalysis, and disease phenotyping provide complementary validation tools to distinguish correlation‐level omics signatures from causality‐supported ferroptosis mechanisms. References in Tables S3−S6 are listed in Table S8. Created with BioRender.com. CPX, ciclopirox olamine; DAB, 3,3'‐diaminobenzidine; HR, hypersensitive response; Lip‐1, liproxstatin‐1; PA, phosphatidic acid; PC, phosphatidylcholine; PE, phosphatidylethanolamine; PEtOH, Phosphatidylethanol; PI, phosphatidylinositol; PS, phosphatidylserine; ROS, reactive oxygen species.

A literature‐based relative host‐pathogen timeline aligns fungal developmental ferroptosis with conidial death, appressorium maturation, and penetration competence, while aligning rice immune ferroptosis with early Ca^2+^/ROS signaling, iron accumulation, ROS amplification, lipid peroxidation, and localized ferroptotic cell death (Figure 2B). The time points are meant as a relative framework across published studies, not as a universal time course for every rice–*M. oryzae* experiment. Based on the public omics databases, a semi‐quantitative pathosystem‐level evidence matrix could be generated (Figure 2C). Evidence strength is assigned according to three criteria: directness of the readout, relevance to infection‐stage ferroptosis, and support from genetic or pharmacological perturbation. Strong support indicates direct omics or biochemical evidence together with perturbation evidence that affects ferroptosis markers and disease or developmental outcomes. Moderate support indicates relevant but partial or indirect evidence, such as module‐level omics changes without full spatial or causal validation. Limited support indicates missing, weak, or mainly inferred evidence. The detailed scoring criteria, source datasets, and supporting references are provided in Tables S3, S6, and S7. Furthermore, the integrated omics signatures could be linked to validation outputs, including lipid ROS, iron staining, fungal biomass, disease lesions, genetic perturbation, and chemical perturbation or rescue (Figure 2D). Overall, the prediction of the ferroptosis regulatory pathway in rice–*M. oryzae* pathosystem based on public multi‐omics data aligns with the existing experimental evidence, demonstrating that integrated omics mining offers a reliable reference for ferroptosis investigation.

## COMPARATIVE BOUNDARY‐SETTING BEYOND RICE BLAST

Although this Commentary is centered on rice–*M. oryzae*, selected systems help define the boundary between ferroptosis and ferroptosis‐like cell death. *Arabidopsis* heat shock remains a useful plant comparison because cell death is associated with ferroptosis characteristics, including iron and ROS dependency and suppression by ferroptosis inhibitors [19]. *Aspergillus flavus* conidial cell death can be induced by Fe^2+^ and attenuated by Nox inhibitors or deletion of *noxA*, supporting a fungal ferroptosis‐like process with a strong ROS component [20]. These comparative systems support the broader relevance of iron‐redox‐lipid death modules, but they should not be presented as equivalent to the rice–*M. oryzae* system in depth. We here summarize these plant and fungal comparisons in Table S1 with information including omics readouts, iron/lipid evidence, and genetic/pharmacological support, also listing the remaining uncertainty.

## FUTURE PERSPECTIVES: FROM OMICS SIGNATURES TO FERROPTOSIS‐GUIDED DISEASE CONTROL

The next phase of ferroptosis research should move from qualitative analyses to spatiotemporal and perturbation‐resolved omics. Iron accumulation or ROS elevation alone is not sufficient, and infection‐associated immune cell death may contain multiple death programs unless ferroptosis‐specific evidence is demonstrated. Moreover, it remains technically challenging to simultaneously resolve ion signals and accurately profile low‐abundance oxidized lipids during host–pathogen interactions. Future studies should combine lipid‐peroxide imaging, spatial lipidomics, metallomics/ionomics, real‐time Fe and Ca^2+^ imaging, genetically encoded redox or calcium sensors, and infection site‐specific transcriptomics/proteomics to determine precise ferroptosis initiation and spatial execution or expansion.

Multi‐omics outcomes must also be tied to causality. Differential transcripts, proteins, or metabolites nominate candidate modules, but they do not prove that a module executes ferroptosis. Causality requires perturbations that shift the omics signatures and phenotypes in the predicted direction. Chemical inhibitors and inducers remain valuable. However, their effects need to be interpreted carefully and in conjunction with mutant analyses, rescue experiments, lipid oxidation assays, and infection phenotyping since Fer‐1, Lip‐1, DFO, CPX, DPI, erastin, EGTA, TFP, and ASM can have dose‐ or context‐dependent effects.

Finally, the functions of ferroptosis in host and pathogen need to be considered individually and in unison prior to its calibrated manipulation for disease control. Suppressing fungal developmental ferroptosis reduces *M. oryzae* pathogenesis, as illustrated by CPX and Lip‐1 treatments. In contrast, tuning rice immune ferroptosis enhances localized resistance, but excessive or mistimed ferroptosis could diminish host fitness. A useful translational strategy is therefore not universal induction or inhibition. Instead, multi‐omics guided identification and exploitation of pathogen‐specific vulnerabilities such as Nox, MoFpn1/autophagy, or PUFA‐PL modules, together with the identification of rice genotypes or treatments that promote spatially confined immune lipid peroxidation, could prove to be more effective in ferroptosis‐based control of rice blast and other important fungal diseases in crops.

In summary, the rice–*M. oryzae* pathosystem provides a rare dual‐organism model in which ferroptosis benefits the pathogen during infection‐related development while aiding the host during immune execution and defense. This Commentary offers an omics‐centered and evidence‐graded framework for ferroptosis regulation in plant–fungal interactions.

## AUTHOR CONTRIBUTIONS

**Zhibin Liang**: Writing—review and editing; writing—original draft; visualization; project administration. **Qing Shen**: Writing—original draft; writing—review and editing; visualization. **Nam Khoa Nguyen**: Visualization; writing—review and editing; writing—original draft. **Ruhui Long**: Writing—original draft; writing—review and editing. **Yuming Ma**: Writing—original draft; writing—review and editing. **Qiao Liu**: Writing—original draft; writing—review and editing. **Naweed I. Naqvi**: Conceptualization; supervision; writing—original draft; writing—review and editing; visualization; project administration. **Nam‐Soo Jwa**: Conceptualization; visualization; writing—review and editing; writing—original draft; project administration; supervision. **Yi‐Zhen Deng**: Writing—original draft; writing—review and editing; visualization; project administration; supervision; conceptualization; funding acquisition.

## CONFLICT OF INTEREST STATEMENT

The authors declare no conflicts of interest.

## ETHICS STATEMENT

No animals or humans were involved in this study.

## Supporting information


**Table S1:** Omics readouts and evidence strength for ferroptosis‐related cell death in plant and fungal systems.
**Table S2:** Rice matrix values for Figure 2A.
**Table S3:** Data, sources, and references for Figure 2A,C.
**Table S4:** Pathogen omics evidence for Figure 2A.
**Table S5:** Pathogen lipidomics for Figure 2A.
**Table S6:** Evidence scoring for Figure 2C.
**Table S7:** Scoring criteria for Figure 2C.
**Table S8:** References in TableS.

## Data Availability

No new experimental datasets were generated in this study. Information on the databases used for interpreting and analyzing ferroptosis characteristics is listed in Supplementary Tables. Supplementary materials (tables, graphical abstract, slides, videos, Chinese translated version, and updated materials) may be found in the online DOI or iMeta Science http://www.imeta.science/.
